# Pharmacokinetics of Locally Applied Antibiotic Prophylaxis for Implant-Based Breast Reconstruction

**DOI:** 10.1001/jamanetworkopen.2023.48414

**Published:** 2023-12-19

**Authors:** Mathilde Nejrup Hemmingsen, Anne Karen Bennedsen, Randa Bismark Kullab, Caroline Barskov Norlin, Mathias Ørholt, Andreas Larsen, Mats Bue, Mads Lichtenberg, Frederik Boetius Hertz, Tine Engberg Damsgaard, Peter Vester-Glowinski, Søren Johannes Sørensen, Thomas Bjarnsholt, Mikkel Herly

**Affiliations:** 1 Department of Plastic Surgery and Burns Treatment, Copenhagen University Hospital, Rigshospitalet, Copenhagen, Denmark; 2 Department of Orthopedic Surgery, Aarhus University Hospital, Aarhus, Denmark; 3 Department of Clinical Medicine, Aarhus University, Aarhus, Denmark; 4 Department of Immunology and Microbiology, University of Copenhagen, Copenhagen, Denmark; 5 Department of Clinical Microbiology, Copenhagen University Hospital, Rigshospitalet, Copenhagen, Denmark; 6 Department of Clinical Medicine, University of Copenhagen, Copenhagen, Denmark; 7 Section of Microbiology, Department of Biology, University of Copenhagen, Copenhagen, Denmark

## Abstract

**Importance:**

Antibiotic irrigation of breast implants is widely used internationally, but no clinical study has investigated the pharmacokinetics of antibiotic prophylaxis in the breast implant pocket.

**Objectives:**

To evaluate how long locally applied gentamicin, cefazolin, and vancomycin concentrations in the implant pocket remain above the minimum inhibitory concentration (MIC) for the most common bacterial infections and to measure systemic uptake.

**Design, Setting, and Participants:**

This prospective cohort study was performed at the Department of Plastic Surgery and Burns Treatment, Rigshospitalet, Copenhagen, Denmark, between October 25, 2021, and September 22, 2022, among 40 patients undergoing implant-based breast reconstruction who were part of the ongoing BREAST-AB trial (Prophylactic Treatment of Breast Implants With a Solution of Gentamicin, Vancomycin and Cefazolin Antibiotics for Women Undergoing Breast Reconstructive Surgery: a Randomized Controlled Trial). Patients were randomized to receive locally applied gentamicin, cefazolin, and vancomycin or placebo. Samples were obtained from the surgical breast drain and blood up to 10 days postoperatively.

**Exposures:**

The breast implant and the implant pocket were irrigated with 160 μg/mL of gentamicin, 2000 μg/mL of cefazolin, and 2000 μg/mL of vancomycin in a 200-mL saline solution.

**Main Outcomes and Measures:**

The primary outcome was the duration of antibiotic concentrations above the MIC breakpoint for *Staphylococcus aureus* according to the Clinical and Laboratory Standards Institute: gentamicin, 4 μg/mL; cefazolin, 2 μg/mL; and vancomycin, 2 μg/mL. Secondary outcomes included the time above the MIC for *Pseudomonas aeruginosa* and other relevant bacteria, as well as systemic uptake.

**Results:**

The study included 40 patients (median age, 44.6 years [IQR, 38.3-51.4 years]; median body mass index, 23.9 [IQR, 21.7-25.9]) with a median number of 3 drain samples (range, 1-10 drain samples) and 2 blood samples (range, 0-6 blood samples). Vancomycin and cefazolin remained above the MIC for *S aureus* significantly longer than gentamicin (gentamicin, 0.9 days [95% CI, 0.5-1.2 days] for blood samples vs 6.9 days [95% CI, 2.9 to 10.9 days] for vancomycin [*P* = .02] vs 3.7 days [95% CI, 2.2-5.2 days] for cefazolin [*P* = .002]). The gentamicin level remained above the MIC for *P aeruginosa* for 1.3 days (95% CI, 1.0-1.5 days). Only cefazolin was detectable in blood samples, albeit in very low concentrations (median concentration, 0.04 μg/mL [range, 0.007-0.1 μg/mL]).

**Conclusions and Relevance:**

This study suggests that patients treated with triple-antibiotic implant irrigation during breast reconstruction receive adequate prophylaxis for *S aureus* and other common implant-associated, gram-positive bacteria. However, the protection against *P aeruginosa* may be inadequate.

## Key Points

**Question:**

For how long after implant-based breast reconstruction with locally applied gentamicin, cefazolin, and vancomycin is the antibiotic concentration in the implant pocket above the minimum inhibitory concentrations (MICs) for the most common bacteria?

**Findings:**

This cohort study of 40 women undergoing implant-based breast reconstruction showed that vancomycin and cefazolin concentrations remained above the MIC for *Staphylococcus aureus* significantly longer than gentamicin (6.9 and 3.7 days vs 0.9 days, respectively). Gentamicin concentrations decreased below the MIC for *Pseudomonas aeruginosa* after 1.3 days.

**Meaning:**

This study suggests that local antibiotic prophylaxis may protect against *S aureus* but may be insufficient against gentamicin-sensitive gram-negative bacteria.

## Teaser

This cohort study evaluates how long locally applied gentamicin, cefazolin, and vancomycin concentrations in the breast implant pocket remain above the minimum inhibitory concentration for the most common bacterial infections and measures systemic uptake.

## Introduction

Breast cancer is one of the most common cancers among women, with approximately 350 000 new cases in the US each year.^1^ Most women undergoing breast reconstruction choose implant-based breast reconstruction,^2,3^ but 5% to 10% of patients develop breast implant infections^4,5,6,7,8^ due to bacterial contamination of the implant either during or after surgery.^9^ Antibiotic treatment is ineffective against implant infections because the bacteria form biofilms on the breast implants.^10,11^ As a result, the infected implants must be surgically removed, and attempts at reimplantation are often postponed for several months. The most common bacterial infections in implant-based breast reconstruction include *Staphylococcus aureus* (23%-35%), followed by other gram-positive bacteria, such as *Staphylococcus epidermidis*, *Streptococcus* species, *Corynebacterium* species, and *Cutibacterium acnes*.^12,13,14,15^ In addition, gram-negative bacteria, including *Pseudomonas aeruginosa*, *Enterobacter cloacae*, *Escherichia coli*, *Klebsiella pneumoniae*, and *Proteus mirabilis*, can cause implant infections.^13,16,17^ Moreover, drug-resistant bacteria are an increasing problem in implant surgery.^18^

To prevent breast implant infections, some surgeons recommend administering prophylactic antibiotics directly onto the breast implant and in the implant pocket,^19,20^ and 70% of US plastic surgeons follow these recommendations,^21^ although the clinical evidence for their effectiveness is sparse.^22^ An in vitro study by Adams et al^19^ suggests that the most effective antibiotic combination against the most common bacterial infections consists of 160 μg/mL of gentamicin, 2000 μg/mL of cefazolin, and 2000 μg/mL of vancomycin.^23^ However, to our knowledge, no clinical study has investigated the antibiotic concentrations in the breast implant pocket, and there is a need for studies investigating the rationale for the choice of antibiotics and doses for optimal prophylaxis.

In this prospective cohort study, we assessed antibiotic concentrations in the implant pocket of patients undergoing implant-based breast reconstruction with locally applied gentamicin, cefazolin, and vancomycin to investigate for how long the prophylactic antibiotic treatment regimen provides protection against bacterial contamination after surgery if the bacteria are susceptible to the antibiotic. This duration was defined as the amount of time above the minimum inhibitory concentration (MIC) for common infective agents, with *S aureus* as the primary benchmark.

## Methods

This prospective observational cohort study, performed between October 25, 2021, and September 20, 2022, was approved by the Regional Committee on Health Research Ethics and the Danish Data Protection Agency. The study was performed according to the Strengthening the Reporting of Observational Studies in Epidemiology (STROBE) reporting guideline^24^ for observational studies. Eligible patients were approached by an investigator prior to the surgery, and they provided written informed consent prior to any study-related activities.

Patients were included from an ongoing randomized clinical trial (the BREAST-AB trial [Prophylactic Treatment of Breast Implants With a Solution of Gentamicin, Vancomycin and Cefazolin Antibiotics for Women Undergoing Breast Reconstructive Surgery: a Randomized Controlled Trial]^25^) of locally applied gentamicin, cefazolin, and vancomycin on breast implants among women undergoing breast reconstruction with implants (ClinicalTrials.gov identifier NCT04731025) at the Department of Plastic Surgery and Burns Treatment, Rigshospitalet, Copenhagen, Denmark. Patients undergoing bilateral breast reconstruction were randomized to receive antibiotics on one side and placebo on the contralateral side as part of the BREAST-AB protocol.^25^ The inclusion criteria were (1) patients undergoing breast reconstruction with permanent implants or in 2 stages with a temporary expander implant, including immediate or delayed reconstruction and flap reconstruction in combination with an implant; (2) participation in the BREAST-AB trial; (3) age older than 18 years; and (4) provision of written informed consent. Patients were excluded if (1) they did not receive surgical drains or (2) the drains were removed before the first sample was obtained. Samples were obtained from both breasts from patients undergoing bilateral surgery regardless of their allocation to antibiotics or placebo to maintain blinding. Data on patient demographic characteristics, surgical characteristics, hospital admission, and drain treatment were obtained prospectively by review of patient medical records according to predefined variables. REDCap, version LTS 13.7.14^26^ was used for data collection.

### Surgical Procedure and Antibiotic Treatment of the Breast Implants

During the surgery, the implant pocket and breast implant were irrigated with 160 μg/mL of gentamicin, 2000 μg/mL of cefazolin, and 2000 μg/mL of vancomycin in 200 mL of saline solution as part of the BREAST-AB trial protocol.^25^ The dissected implant pocket was soaked in 150 mL of the solution while the breast implant was soaked in 50 mL of the solution for a minimum of 5 minutes before implantation. The excess fluid from the dissected implant pocket was removed using suction. Any other treatment adhered to the department’s standard treatment, which included intravenous perioperative cefuroxime. After surgery, 2 surgical drains were placed in each breast implant pocket: 1 draining the area near the inframammary fold and 1 draining the area close to the axilla. The drains were kept in place until fluid production decreased to less than 30 mL/d.

### Sampling Procedure and Analysis of Antibiotic Concentrations

Drain fluid and venous blood samples were collected simultaneously at random time points after surgery until the drains were removed as part of the standard treatment. The drain samples were collected at random time points that varied among patients to obtain high-resolution concentrations over time for the entire patient population. Before sampling, the volume of fluid in the drain container was registered and discarded. Then, 10 mL of the newly formed fluid was aspirated for analysis from the drain tube. As a standard, the drain sample was obtained from the inframammary drain. After anonymization, samples from the breasts that received placebo were discarded by an unblinded member of the coordinating trial unit of the BREAST-AB trial. In addition, a venous blood sample was obtained at the time of the drain sample. The blood and drain samples were collected in EDTA tubes, kept at 5 °C, and centrifuged within 30 minutes at a speed of 3000*g* for 10 minutes at 4 °C. Then, 2 mL of the supernatant from the drain fluid and plasma from the blood sample were pipetted into individual low-protein–binding tubes and kept at −80 °C until analysis.

The concentrations of gentamicin, cefazolin, and vancomycin were analyzed using high-performance liquid chromatography–mass spectrometry with a limit of quantitation of 0.05 μg/mL. Calibration and quality control samples were prepared in blank human plasma and 0.1% saline, respectively. Benzylpenicillin was used as an internal standard, and plasma samples were precipitated by using 250 μg/mL of plasma on a 30-kDa molecular weight cutoff filter followed by dilution of the filtrate. For a more detailed description of the sample analysis, see the eMethods in Supplement 1.

### Main Outcome Measures

The primary outcome was the duration of time that the antibiotic concentrations in the implant pocket were above the MIC for *S aureus.* The MIC was defined as the clinical breakpoint of *S aureus* according to the Clinical and Laboratory Standards Institute *M100 Performance Standards for Antimicrobial Susceptibility Testing*, 33rd edition^27^: gentamicin, 4 μg/mL; cefazolin, 2 μg/mL; and vancomycin, 2 μg/mL. We chose the time above MIC as the primary target because systemic pharmacokinetic measurements, such as the area under the curve above the MIC, do not necessarily apply to the high antibiotic levels in tissue that are achieved with locally applied antibiotics.^28^ We chose *S aureus* as the primary benchmark for each antibiotic because it is the most common type of bacterial infection in breast implants^14^ and because the MIC of *S aureus* is high compared with that of other relevant gram-positive bacteria. Secondary outcomes included the duration of time above the MIC for *S epidermidis*, *Streptococcus* species, *Corynebacterium* species, *C acnes*, *P aeruginosa*, *E cloacae*, *E coli*, *K pneumoniae*, and *P mirabilis* (the MICs for each antibiotic are listed in the eTable in Supplement 1). Additional secondary outcomes included the peak concentration and antibiotic half-life in the breast implant pocket for the 3 antibiotics and the systemic uptake of the locally applied antibiotics assessed by antibiotic concentrations in venous blood samples.

### Statistical Analysis

According to prelimnary data from 11 included patients (written communication with K. Borchert, Bioxpedia A/S, March 7, 2022), 40 patients were required to estimate the antibiotic concentration in the implant pocket after 72 hours with a margin of error of 2 μg/mL and an SD of 6.5 μg/mL. Descriptive statistics were used to present baseline characteristics. Binary outcomes were described as simple proportions, and continuous outcomes were described as mean (SD) values or median (IQR) values, depending on the distribution. For each antibiotic, the concentration over time was modeled as a 3-parameter Weibull dose-response function with a fixed lower limit of zero and with a robust covariance matrix estimation to account for multiple measurements per patient.^29^ Model selection was performed as a comparison of Weibull, log-logistic, quadratic, and cubic models, and the final model was chosen based on the lowest value of the Akaike information criterion. The following parameters were calculated for each curve: peak concentration, antibiotic half-life, and time above the MIC. Comparisons of times above the MIC and antibiotic half-lives for gentamicin, cefazolin, and vancomycin were tested with *z* score tests and reported as Bonferroni-adjusted *P* values. All statistical tests were 2-sided, and *P* < .05 was considered statistically significant except for cases of multiple testing, where a Bonferroni correction was applied. All analyses and plots were performed in R software, version 4.2.2 (R Project for Statistical Computing), with the packages tidyverse, drc, and PKNCA.^30,31,32^

## Results

Between October 25, 2021, and September 20, 2022, 40 patients (median age, 44.6 years [IQR, 38.3-51.4 years]; median body mass index [calculated as weight in kilograms divided by height in meters squared], 23.9 [IQR, 21.7-25.9]) undergoing implant-based breast reconstruction met the inclusion criteria and provided written informed consent for participation in the study (Table 1). All 40 patients received surgical drains during surgery, and none of the patients were excluded from the study due to premature removal of surgical drains. The included patients contributed 146 drain samples with a median number of 3 drain samples per patient (range, 1-10 drain samples per patient) collected between 0.6 and 239.5 hours (approximately 0.03-10.0 days) after surgery. During this period, the median daily drain output per patient was 47.5 mL (IQR, 29.3-69.8 mL). In addition, we collected 66 blood samples from 26 of 40 patients (65%) between 0.8 and 117.6 hours (approximately 0.03-4.9 days) after surgery (median, 2 blood samples [range, 0-6 blood samples]). Fourteen patients did not wish to contribute blood samples. Table 1 describes the baseline characteristics of the 40 included patients.

**Table 1.** Baseline Characteristics of the Patients

Characteristic	Value
No. of patients	40
No. of breasts	40
Right breast, No. (%)	18 (45)
Left breast, No. (%)	22 (55)
Laterality of breast reconstruction, No. (%)	
Bilateral	27 (68)
Unilateral	13 (33)
Age, median (IQR), y	44.6 (38.3-51.4)
Height, median (IQR), cm	168 (165-169)
Weight, median (IQR), kg	67.0 (61.5-75.0)
BMI, median (IQR)	23.9 (21.7-25.9)
Timing of breast reconstruction, No. (%)	
Immediate	37 (93)
Delayed	3 (8)
Type of breast reconstruction, No. (%)	
Direct to permanent implant	29 (73)
2-Stage with an expander implant	9 (23)
Latissimus dorsi flap reconstruction in combination with an implant	2 (5)
Tissue plane used for the implant pocket, No. (%)	
Prepectoral	31 (78)
Subpectoral	9 (23)
Total No. of drain samples	146
No. of drain samples per patient, median (IQR) [range]	3 (3-4) [1-10]
Time from antibiotic administration to drain sampling, median (IQR) [range], h	44.0 (23.6-68.2) [0.6-239.5]
Daily drain output volume per patient, median (IQR) [range], mL	47.5 (29.3-69.8) [9.0-243.0]
Total No. of blood samples	66
No. of blood samples per patient, median (IQR) [range]	2 (0-3) [0-6]
Time from antibiotic administration to blood sample, median (IQR, range), h	32.3 (19.7-50.8) [0.8-117.6]
Length of hospital stay, median (IQR) [range], d	3.1 (2.7-3.9) [1.9-10.1]

Abbreviation: BMI, body mass index (calculated as weight in kilograms divided by height in meters squared).

### Duration of Inhibitory Concentrations

The concentration of vancomycin in the implant pocket remained above the MIC for *S aureus* for 6.9 days (95% CI, 2.9-10.9 days) compared with 3.7 days (95% CI, 2.2-5.2 days) for cefazolin and 0.9 days (95% CI, 0.5-1.2 days) for gentamicin (Figure and Table 2). This period was statistically significantly shorter for gentamicin than for vancomycin (*P* = .02) and cefazolin (*P* = .002). Similarly, compared with gentamicin, the vancomycin concentration was higher than the MIC for *S epidermidis* for a significantly longer period of time (vancomycin, 5.9 days [95% CI, 2.9-8.8 days]; gentamicin, 0.9 days [95% CI, 0.5-1.2 days]; *P* = .006). The vancomycin concentration exceeded the MIC for *Corynebacterium* species, such as *C striatum* and *C amycolatum*, for 9.0 days (95% CI, 2.5-15.5 days) and exceeded the MIC for *Streptococcus* species, such as *S pyogenes* and *S agalactiae*, for 8.0 days (95% CI, 2.8-13.1 days), although the duration of time above the MIC for the *Streptococcus* species was not statistically significantly longer than that of cefazolin (5.8 days [95% CI, 2.3-9.4 days]; *P* ≥ .99). In contrast, the gentamicin concentration above the MIC for gram-negative bacteria, such as *P aeruginosa* and *E cloacae*, lasted only 1.3 days (95% CI, 1.0-1.5 days). The duration of cefazolin concentrations above the MIC for *E coli*, *K pneumoniae*, and *P mirabilis* lasted for 3.2 days (95% CI, 2.1-4.3 days), which was significantly longer than that of gentamicin (1.3 days [95% CI, 1.0-1.5 days]; *P* = .006). The duration of antibiotic concentrations above the MIC for each bacterium is shown in Figure and Table 2.

**Figure.**
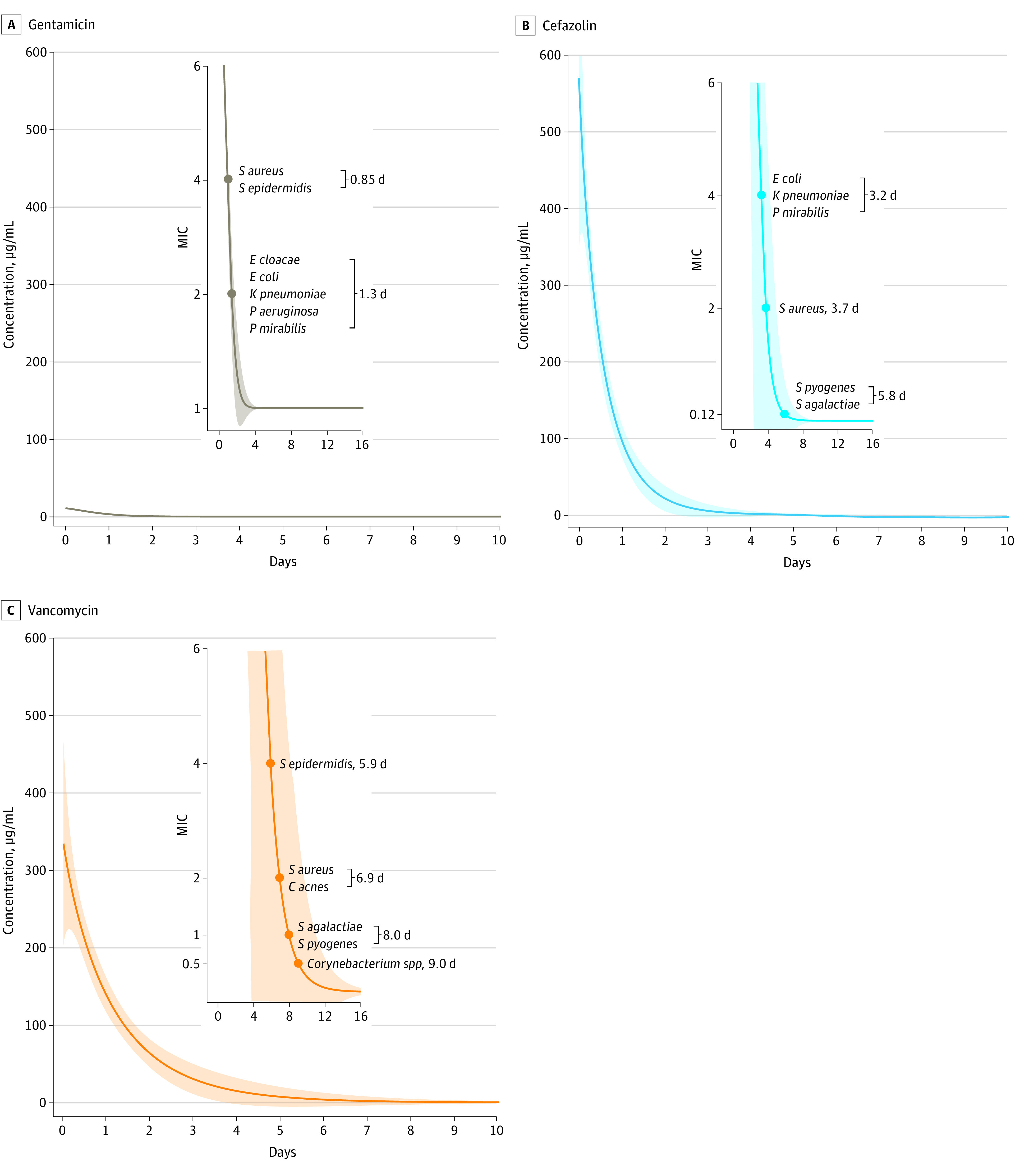
Concentrations of Gentamicin, Cefazolin, and Vancomycin in Drain Fluid Samples From the Implant Pocket Over Time *C acnes* indicates *Cutibacterium acnes*; *E cloacae*, *Enterobacter cloacae*; *E coli*, *Escherichia coli*; *K pneumoniae*, *Klebsiella pneumoniae*; *P aeruginosa*, *Pseudomonas aeruginosa*; *P mirabilis*, *Proteus mirabilis*; *S agalactiae*, *Streptococcus agalactiae*; *S aureus*, *Staphylococcus aureus*; *S epidermidis*, *Staphylococcus epidermidis*; and *S pyogenes*, *Streptococcus pyogenes*. Shaded areas indicate 95% CIs.

**Table 2.** Duration of Time Above MIC for Gentamicin, Cefazolin, and Vancomycin

Bacteria	Effective antibiotics	Antibiotic with the longest duration of time above MIC	Duration of time above MIC (95% CI), d
Gram-positive bacteria			
* Staphylococcus aureus*	Gentamicin, cefazolin, vancomycin	Vancomycin	6.9 (2.9-10.9)
* Staphylococcus epidermidis*	Cefazolin, vancomycin	Vancomycin	5.9 (2.9-8.8)
* Streptococcus agalactiae*	Cefazolin, vancomycin	Vancomycin	8.0 (2.8-13.1)
* Streptococcus pyogenes*	Cefazolin, vancomycin	Vancomycin	8.0 (2.8-13.1)
* Corynebacterium striatum*	Vancomycin	Vancomycin	9.0 (2.5-15.5)
* Corynebacterium amycolatum*	Vancomycin	Vancomycin	9.0 (2.5-15.5)
* Cutibacterium acnes*	Vancomycin	Vancomycin	6.9 (2.9-10.9)
Gram-negative bacteria			
* Pseudomonas aeruginosa*	Gentamicin	Gentamicin	1.3 (1.0-1.5)
* Enterobacter cloacae*	Gentamicin	Gentamicin	1.3 (1.0-1.5)
* Escherichia coli*	Gentamicin, cefazolin	Cefazolin	3.2 (2.1-4.3)
* Klebsiella pneumoniae*	Gentamicin, cefazolin	Cefazolin	3.2 (2.1-4.3)
* Proteus mirabilis*	Gentamicin, cefazolin	Cefazolin	3.2 (2.1-4.3)

Abbreviation: MIC, minimum inhibitory concentration.

### Antibiotic Half-Life and Peak Concentration in the Implant Pocket

The half-lives of vancomycin and gentamicin in the breast implant pocket were similar (gentamicin, 0.65 days; vancomycin, 0.74 days), while the half-life of cefazolin was shorter (0.34 days), although the difference was not statistically significant (*P* = .06). The peak gentamicin concentration measured using drain fluid samples from the implant pocket was 10.8 μg/mL (6.8% of the administered dose). This concentration was contrasted by cefazolin and vancomycin, with peak concentrations of 607 μg/mL (30.4% of the administered dose) and 344 μg/mL (17.2% of the administered dose), respectively, even though all 3 antibiotics were measured from the same drain fluid.

### Systemic Uptake of Locally Applied Antibiotics

Locally applied gentamicin and vancomycin were not detected in any of the blood samples. Cefazolin was detected in 6 of 66 blood samples (6 of 26 patients), with a median cefazolin concentration of 0.04 μg/mL (range, 0.007-0.1 μg/mL) 5 to 8 hours after local application on the breast implant.

## Discussion

This study found that prophylactic local antibiotic treatment for implant-based breast reconstruction may provide approximately 7 days of protection against the most common types of bacterial infection. We found that the concentration of vancomycin in the breast implant pocket remained above the MIC for *S aureus* for approximately 7 days, while this period lasted only 0.9 days for gentamicin and 3.7 days for cefazolin. However, the protection against gram-negative bacteria such as *P aeruginosa* and *E cloacae*, which solely relies on gentamicin in the antibiotic prophylactic treatment regimen, lasted for only 1.3 days. The concentration of cefazolin remained above the MIC for 3.2 days against the gram-negative bacteria *E coli*, *K pneumoniae*, and *P mirabilis*.

To our knowledge, measurements of antibiotic concentrations in the breast implant pocket after local application of antibiotic prophylaxis have not been performed, and the duration of protection is expected to be brief.^33^ This expected duration is in contrast to the findings of this study, which showed a long-lasting concentration of vancomycin, and to some extent cefazolin, in the implant pocket. This is an important finding because studies suggest that bacterial contamination of the implant and formation of bacterial biofilm^34^ can take place after implant-based breast reconstruction surgery.^9^

In a previous study, White et al^35^ found that locally applied cefazolin for patients undergoing breast reduction without the use of an implant remained above the MIC for *S aureus* (8 μg/mL) in surgical breast drains for 24 hours. However, that study measured the concentrations only up to 24 hours and therefore could not estimate the total period of a concentration that would inhibit *S aureus*. Moreover, the pharmacokinetics would most likely be affected by the presence of an implant.

The short duration of gentamicin concentrations above the MIC for *P aeruginosa* and *E cloacae*, which lasted only 1.3 days, cannot be explained by the elimination half-life because the half-lives of gentamicin and vancomycin were similar. An explanation could be the differences in how the antibiotics are resorbed by the soft tissue around the implant. However, a more likely explanation is that the administered dose of gentamicin was only 80× MIC for *P aeruginosa* (2 μg/mL), whereas the dose of vancomycin was 1000× MIC for *S aureus* (2 μg/mL), which may explain why vancomycin maintained an effective concentration for significantly longer than gentamicin. We hypothesize that an increase in the gentamicin dose (eg, to a level similar to that of vancomycin) would prolong the duration of gentamicin concentrations above the MIC in the implant pocket and thereby potentially provide longer protection from gram-negative bacteria such as *P aeruginosa* and *E cloacae.* However, multiple factors may be associated with the elimination of antibiotics from the implant pocket, and future studies are needed to investigate whether an increased dose would provide a prolonged duration of antibiotic concentrations above the MIC and whether this, in turn, would lead to fewer implant infections.

We found that the systemic uptake of the locally applied antibiotics was minimal, which is in line with a previous study.^35^ We did not detect any gentamicin in the blood samples after the local application of 160 μg/mL of gentamicin, suggesting that the dose of gentamicin could be increased in future studies without risking systemic adverse effects. However, further studies are needed to assess the risk of local and systemic adverse effects of an increased dose of gentamicin. Only cefazolin was detectable in the blood samples and only in very low concentrations ranging from 0.007 to 0.1 μg/mL. These concentrations are extremely low compared with peak cefazolin serum concentrations of approximately 100 μg/mL after intravenous administration of 0.5 g of cefazolin,^36^ which makes systemic adverse effects very unlikely.

### Limitations

This study has several limitations. First, we measured the antibiotic concentration in the breast implant pocket by obtaining fluid samples from the surgical drains. This creates a latency because of the small reservoir of fluid in the drain tubes, which is a potential problem for patients with very low drain fluid production. However, we do not believe that this would have had any significant association with our results because the median drain production in this study was 47.5 mL/d and drain tubes can contain only up to a few milliliters. Second, only 26 of 40 patients opted to provide blood samples, but given the negligible levels of antibiotics in the blood samples, we do not believe that this has limited the conclusions of the study. A third limitation is that we did not adjust the antibiotic concentrations for drain output volume per day because the antibiotic irrigation is administered only as a single dose and before the postoperative drain output is known. Therefore, it would not be possible to adjust the doses according to the drain output of the individual patient. A fourth limitation is that we did not stratify the patients according to the subtype of breast reconstruction or patient characteristics. This was not possible because of limited statistical power, although it could have potentially influenced the antibiotic concentration in the implant pocket. Larger studies are needed to investigate how the surgical technique and patient characteristics are associated with the antibiotic concentration in the breast implant pocket. Fifth, this study investigated only how long the antibiotic concentrations in the implant pocket remained above the MIC for the most common bacterial infections in cases of implant infection and not whether this period was sufficient to prevent implant infections. Thus, further studies are needed, including the results from an ongoing randomized placebo-controlled clinical trial^25^ (ClinicalTrials.gov identifier NCT04731025) that will determine whether local application of the triple-antibiotic solution leads to fewer implant infections compared with placebo.

## Conclusion

In this cohort study, the concentration of vancomycin in the implant pocket after a single dose of locally applied gentamicin, vancomycin, and cefazolin provided protection against *S aureus* for approximately 7 days, while the protection against gentamicin-susceptible bacteria, such as *P aeruginosa*, lasted only 1.3 days. The systemic uptake of the locally applied antibiotics was negligible, which suggests a low risk of systemic adverse effects from the treatment.

## Notes

Supplement 1.
eMethods.
**eTable.** MIC of the Common Bacteria Associated With Implant Infections According to the CLSI M100-ED33:2023 Performance Standards for Antimicrobial Susceptibility Testing, 33rd Edition

Supplement 2.
Data Sharing Statement
