# Supplementary material for: Pharmacokinetics of Locally Applied Antibiotic Prophylaxis for Implant-Based Breast Reconstruction
Source: JAMA Netw Open. 2023 Dec 19;6(12):e2348414. doi: 10.1001/jamanetworkopen.2023.48414 (PMC10731505; doi:10.1001/jamanetworkopen.2023.48414)
Supplement: Supplement 1. — eMethods. eTable. MIC of the Common Bacteria Associated With Implant Infections According to the CLSI M100-ED33:2023 Performance Standards for Antimicrobial Susceptibility Testing, 33rd Edition [file jamanetwopen-e2348414-s001.pdf]

Supplementary Online Content

Hemmingsen MN, Bennedsen AK, Kullab RB, et al. Pharmacokinetics of locally applied antibiotic prophylaxis for implant-based breast reconstruction. *JAMA Netw Open.* 2023;6(12):e2348414. doi:10.1001/jamanetworkopen.2023.48414

eMethods.

eTable. MIC of the Common Bacteria Associated With Implant Infections According to the CLSI M100-ED33:2023 Performance Standards for Antimicrobial Susceptibility Testing, 33rd Edition

This supplementary material has been provided by the authors to give readers additional information about their work.

© 2023 Hemmingsen MN et al. *JAMA Network Open.*

eMethods. A detailed description of the analysis method for quantification of antibiotic concentrations in drain fluid and plasma samples.

The plasma and drain fluid concentrations of cefazolin, gentamicin, and vancomycin were analyzed using High-Performance Liquid Chromatography-Mass Spectrometry (HPLC-MS/MS). Calibration curves for all analytes were prepared at 100, 50, 25, 10, 5, and 1 µg/mL solutions by adding stock solutions to 0.1% NaCl and blank human plasma, respectively. The quality controls were prepared in blank human plasma and in 0.1% NaCl (80, 45, and 5 µg/mL). The concentration in plasma was measured by filtrating 250 µL plasma on a 30 kDa Molecular Weight Cut-Off (MWCO) filter obtained from Pall Corporation. The samples were spun at 2000G for 30 minutes. Then, 10 µL filtrate was moved to a new deep well plate, and 490 µL H2O with 200 ng/mL benzylpenicillin was added to each well. Blank human plasma was used for blanks, while pure LC-MS grade H2O was used for double blanks. After dilution, a pierceable silicone lid was placed on top of the plate, after which the plate was vortexed for 10 seconds and centrifuged for 5 minutes at 500G. The plates were put directly into the autosampler to perform the analysis.

The HPLC-MS/MS system consisted of an Exion LC AD HPLC system coupled to a Sciex 4500 qTrap equipped with an electrospray ionization source. Separation took place on a Phenomenex Kinetex XB-C18 2,6 µm 50x2,1 mm 100 Å at 65 °C. The mobile phases had a flow rate of 0.3 mL/min and were composed of (A) LC-MS grade H_2O with 0.1 % formic acid; and (B) LC-MS acetonitrile with 0.1 % formic acid.

The gradient profile was: 10% B to 95%B from 0 to 3.90 min, 95% B from 3.9 to 3.95 min, 95% B to 10% B from 3.95 min to 4.6 min, and ending with 10 % B followed by gradient starting conditions and re-equilibration for 1 min, resulting in a total run time of 5.6 min per sample. The analytes

© 2023 Hemmingsen MN et al. *JAMA Network Open.*

were detected by positive electrospray ionization (ESI) mode using multiple reaction monitoring (MRM). The cefazolin concentrations were quantified using the mass transition m/z 455.02 → 322.75 as a quantifier and 455.02 → 156.1 as a qualifier. The gentamicin concentrations were quantified using the mass transition m/z 478.4.0 → 322.2 as a quantifier and 464.4 → 322.2 and 450.4 → 322.2 as a qualifier. The vancomycin concentrations were quantified using the mass transition m/z 725.4 → 144.0 as a quantifier. No qualifier was found. Source/Gas parameters were set to the following: curtain gas: 30; collision gas: medium; ion spray voltage: 5500V; temperature: 400 C°; ion source gas 1: 40; and ion source gas 2: 40. CV% was calculated with three replicas of spiked plasma at concentration levels of 800, 450, and 70 ng/mL. All CV% were below 20%.

© 2023 Hemmingsen MN et al. *JAMA Network Open.*

eTable. MIC of the Common Bacteria Associated With Implant Infections According to the CLSI M100-ED33:2023 Performance Standards for Antimicrobial Susceptibility Testing, 33rd Edition

**Bacteria**	**MIC (µg/mL)**		
	**Gentamicin**	**Vancomycin**	**Cefazolin**
Staphylococcus aureus	4	2	2
Staphylococcus epidermidis	4	4	-
Streptococcus agalactiae	-	1*	0.12*
Streptococcus pyogenes	-	1*	0.12*
Corynebacterium striatum	-	0.5	-
Corynebacterium amycolatum	-	0.5	-
Cutibacterium acnes	-	2	-
Pseudomonas aeruginosa	2**	-	-
Enterobacter cloacae	2	-	-
Escherichia coli	2	-	4
Klebsiella pneumoniae	2	-	4
Proteus mirabilis	2	-	4

*Also covers Viridans *streptococci* such as *S mutans* which have been seen to cause bacterial biofilm on implants.
**According to the CLSI M100-ED33:2020 Performance Standards for Antimicrobial Susceptibility Testing, 30th Edition.

© 2023 Hemmingsen MN et al. *JAMA Network Open.*
