# Supplementary material for: Pharmacokinetics of Locally Applied Antibiotic Prophylaxis for Implant-Based Breast Reconstruction
Source: JAMA Netw Open. 2023 Dec 19;6(12):e2348414. doi: 10.1001/jamanetworkopen.2023.48414 (PMC10731505; doi:10.1001/jamanetworkopen.2023.48414)
Supplement: Supplement 2. — Data Sharing Statement [file jamanetwopen-e2348414-s002.pdf]

Data Sharing Statement

Hemmingsen. Pharmacokinetics of Locally Applied Antibiotic Prophylaxis for Implant-Based Breast Reconstruction. *JAMA Netw Open*. Published December 19, 2023.
doi:10.1001/jamanetworkopen.2023.48414

Data
Data available: Yes
Data types: Deidentified participant data
How to access data: Access to data upon resonable request can be sent to mathilde.nejrup.hemmingsen@regionh.dk
When available: With publication

Supporting Documents
Document types: None

Additional Information
Who can access the data: Researchers whose proposed use of the data has been approved
Types of analyses: For any purpose
Mechanisms of data availability: With investigator support
